# Hybrid Pectin-Liposome Formulation against Multi-Resistant Bacterial Strains

**DOI:** 10.3390/pharmaceutics12080769

**Published:** 2020-08-14

**Authors:** Lígia Nunes de Morais Ribeiro, Eneida de Paula, Daise Aparecida Rossi, Guilherme Paz Monteiro, Edson Campos Valadares Júnior, Rogério Reis Silva, Rodrigo Rodrigues Franco, Foued Salmen Espíndola, Luiz Ricardo Goulart, Belchiolina Beatriz Fonseca

**Affiliations:** 1 School of Veterinary Medicine, Federal University of Uberlândia, Uberlândia 38402-018, Brazil; daise.rossi@ufu.br (D.A.R.); guil.paz@hotmail.com (G.P.M.); edson2campos@hotmail.com (E.C.V.J.); rogerioreissilva98@gmail.com (R.R.S.); biafonseca@ufu.br (B.B.F.); 2 Department of Biochemistry and Tissue Biology, Institute of Biology, University of Campinas, Campinas 13083-862, Brazil; depaula@unicamp.br; 3 Institute of Biotechnology, Federal University of Uberlândia, Uberlândia 38405-319, Brazil; rodrigorfr@yahoo.com.br (R.R.F.); fsespindola@gmail.com (F.S.E.); lrgoulart@ufu.br (L.R.G.)

**Keywords:** drug delivery, hybrid formulations, liposomes, biopolymers, fluoroquinolones, multidrug resistance

## Abstract

This work describes the development of a gastroresistant antimicrobial formulation composed of two carriers, pectin and liposomes, intended to improve the efficiency of norfloxacin (NOR) against multi-resistant bacterial strains. The formulations showed physicochemical stability for 180 days (4 °C) in terms of size, polydispersity, and zeta potential of the vesicles, prolonging the in vitro release of NOR for 11 h. The hybrid nanocarriers improved the in vitro antimicrobial activity against different multidrug-resistant bacterial strains, such as *Salmonella* sp., *Pseudomonas* *aeruginosa*, *E. coli* and *Campylobacter* *jejuni*, in comparison to commercial NOR and liposomal suspensions. The in vivo toxicity assay in chicken embryos revealed that the hybrid systems were not toxic in any of the different parameters analyzed, a result also corroborated by the analyses of biochemical biomarkers of the chicken-embryos liver function.

## 1. Introduction

Antibiotics are among the most important class of drugs employed to minimize mortality caused by bacterial infections, also preventing complications in medical and veterinary invasive interventions [1]. In the past years, a huge increase in multidrug resistant bacterial strains was noticed, mainly due to the indiscriminate use of antibiotic agents, being a severe concern in veterinary science and medicine [2]. Norfloxacin (NOR, Figure S1) is a third-generation quinolone antibiotic commonly administered in serious bacterial infections caused by Gram-positive and Gram-negative strains. However, NOR is an amphoteric molecule of poor water solubility and low bioavailability in humans and animals post oral administration (<40%), which contributed to limited antimicrobial activity and microorganisms resistance [3]. These physicochemical properties make its use often ineffective by the oral route, allied to reports of gastrointestinal and bowel side effects [4].

Lately, novel therapies based on nanostructured drug delivery systems have been proposed, in order to improve the antimicrobial activity of traditional antibiotics, without compromising its safety. Liposomal antibiotic formulations date back 50 years, given the ability of the lipid vesicles to delivery antibiotics on the specific targets, being useful against several bacterial infections [5]. The incorporation of the antibiotic molecule in the vesicle may occur in the lipid bilayer and/or in its inner aqueous compartment, so that the drug gets protected from degradation, prolongs its release kinetics and produces less side effects [6]. Several classes of antibiotics have been successfully encapsulated in liposomes (LIP) for multiapplication [7], including NOR, pefloxacin and ofloxacin [8,9].

Organic–organic nanostructured delivery systems are hybrid pharmaceutical forms composed of at least two different organic matrices: protein–polymer, protein–lipid, polymer–polymer or lipid–polymer [10]. This strategy combines the advantage of each excipient in a single system, providing specialized nanodevices for the specific interaction with the biological barrier of interest [11]. Among the nanohybrid systems, lipid–polymer nanoparticles and hybrid hydrogels are the most frequent systems reported in the literature for the sustained release of anesthetics, antineoplastics and antibiotics [12,13,14]. Pectin (PCT, Figure S2) is a biocompatible anionic heterosaccharide present in the cell wall of citrus fruits. It has been widely described as a biopolymer matrix for drug-delivery systems [15,16] due to its resistance to low pH medium and the ability to interact with mucin, the major glycoprotein of mucous tissues.

In this work, nanohybrid formulations based on liposomes and PCT were investigated, in order to improve the antimicrobial activity of NOR (0.2% *w*/*v*) against multi-resistant bacteria and to protect the gastrointestinal tract after oral administration. Conventional liposomes containing or not NOR (LIP/NOR, LIP, respectively) were prepared and compared with two types of hybrid systems: (i) liposomes plus PCT solution (PCT-LIP/NOR, PCT-LIP), a blend in which the polymer was only in the outer aqueous phase of the vesicles prepared at pH 7.0, and (ii) liposomes containing PCT in the inner/out aqueous phase (PCT@LIP/NOR, PCT@LIP), prepared without buffer solution, at pH 4.8. The size (nm), polydispersity (PDI), and zeta potential (mV) of the particles were followed in a stability study, for 180 days (4 °C). NOR encapsulation efficiency and in vitro release profile were quantified by UV-vis absorption. The antimicrobial activity of the hybrid formulations was determined against *Salmonella* Heildelberg, *Salmonella* Typhimuirium, *Campylobacter jejuni*, *Pseudomonas aeruginosa* and Avian Pathogenic *Escherichia coli* (APEC) multi-resistant strains, and the safety of the hybrid formulations was confirmed through the in vivo embryo chicken-test model.

## 2. Material and Methods

### 2.1. Materials

Egg phosphatidylcholine (EPC), cholesterol (Chol), α-tocopherol, pectin from apple (PCT) and norfloxacin were purchashed by Sigma-Aldrich (St Louis, MO, USA). Deionized water (18 MΩ) was obtained from an Elga USF Maxima Ultra-Pure water purifier (Elga LabWater, High Wycombe, UK). All other reagents were of analytical or pharmaceutical grade.

### 2.2. Preparation of Hybrid Liposomal Formulations

A chloroform solution of EPC, Chol and α tocopherol at 4:3:0.07 mol % [17] was dried under N_2_ flux and vacuum for 2 h for solvent evaporation. The lipid film was then hydrated in 10 mM HEPES buffer pH 7.4 containing 2 mg/mL norfloxacin (LIP/NOR) or not (control, LIP). Thus, the samples were vortexed for 3 min and extruded (12 cycles) through porous (400 nm wide) polycarbonate membranes to produce large unilamellar or oligolamellar vesicles. Two types of hybrid systems were prepared: (1) PCT-LIP/NOR hybrid system and its control (PCT-LIP), in which an aqueous solution of pectin (1%, *w*/*v*) was added (1:1 *v:v*) to the already formed liposomes, containing or not NOR, resulting in a final 0.5% PCT concentration. The samples were left under magnetic stirring for 30 min prior to vortexing and extrusion; (2) PCT@LIP/NOR and its control (PCT@LIP) system, where—instead of HEPES buffer—0.5% PCT solution, with or without 0.2% NOR, was used to hydrate and resuspend the lipid film prior to liposomes formation. After vortexing and extrusion, liposomes of controlled size and lamellarity were prepared, as described above. The total lipid concentration in all liposome formulations (LIP/NOR, LIP, PCT-LIP/NOR, PCT-LIP, PCT@LIP/NOR, and PCT@LIP) was set to 10 mM.

### 2.3. Physicochemical Stability

The average particle size (nm), polydispersion index (PDI) and zeta potential (mV) of the liposomes, with and without 0.2% NOR (*w*/*v*) were monitored for 180 days, using dynamic light scattering (DLS) in a ZetaSizer ZS90 (Malvern Instruments, Malvern, UK). The measurements were carried out in triplicate (25 °C) and ANOVA/Tukey tests were employed (*p* < 0.05) for statistical analysis.

### 2.4. Morphology of Hybrid Liposomes

Elucidation of the morphology of hybrid system, with or without NOR was assessed by scanning electron microscopy by field emission (FE-SEM) and transmission electron microscopy (TEM) analyses. For FE-SEM, a drop of the sample was adhered to a stub. After complete solvent evaporation, the stubs were sputtered with gold bath for 120 s at 30 kV. The samples were visualized in a field JEOL electron scanning microscope (model JSM 5800LV), operating under a variable voltage from 0.3 to 30 kV, with tungsten filament, using the SemAfore 5.21 image capture system software. For TEM images, a drop of the sample was added to a copper grid. After 60 s, the excess sample was removed with filter paper. Then, a drop of (2% *w*/*w*) uranyl acetate was added, to provide contrast. After 60 s in a dark field, the excess liquid was removed, and, subsequently, a drop of deionized water was added to the grid and the excess was subsequently removed. The samples were visualized in a Zeiss–LEO 906 TEM, operating at 60 kV and equipped with an Olympus iTEM CCD camera and image capture software.

### 2.5. Encapsulation Efficiency (%EE)

The encapsulation efficiency (%EE) of NOR by the formulations was determined by the ultrafiltration-centrifugation method at 12 °C (4000× *g* 6 min), through 10 kDa porous regenerated cellulose filters (Millex, Millipore, Burlington, MA, USA). The amount of NOR was quantified by UV-vis absorption (λ = 275 nm) and %EE was calculated according to Equation (1):

(1) $$\%\mathrm{EE}=\;\frac{\left(\mathrm{total}\;\mathrm{NOR}\;–\;\mathrm{free}\;\mathrm{NOR}\right)}{\mathrm{total}\;\mathrm{NOR}\;}\times 100$$

where: total NOR is the total drug concentration and free NOR is the non-encapsulated fraction of the drug, determined in the filtrate.

### 2.6. In Vitro Release Test

The in vitro release profile of NOR, in solution (free) or encapsulated in the liposomes (LIP/NOR, PCT-LIP/NOR and PCT@LIP/NOR) was performed by dialysis method through (10,000 Da molecular exclusion pores) dialysis bags. The dialysis systems were immersed in 10 mM PBS pH 7.4 containing 2% Tween 80 (*w*/*v*). The samples were kept under magnetic stirring at 350 rpm, 37 °C for 11 h, ensuring sink conditions. At determined intervals, the aliquots were spectrophotometrically quantified at 275 nm, and immediately replaced in order to keep the sample volume constant. The absorbance measurements were converted into a percentage of NOR released as a function of time (*n* = 5). The kinetic curves modeling was carried out through KinetDS 3.0 software (Aleksander Mendyk, Kraków, Poland) [18]. Several models were tested (zero order, first order, Higuchi, Korsmeyer-Peppas and Weibull). Korsmeyer-Peppas (Equation (2)) described better the kinetics of LIP/NOR and PCT-LIP/NOR samples, while the first order model (Equation (3)) was the best fitted model (Table S1) for PCT@LIP/NOR, considering the coefficient of determination (R^2^).

(2) $$Q=k.t^{n}$$

*ln* [1 − (Mt/M∞)] − kt (3)

where: *Q* is the amount of drug released at time t, k is a constant, *n* is the release exponent [19] in Korsmeyer-Peppas equation, while M_t_/M∞ is the drug fraction released at time *t*, in Equation (3) [20].

### 2.7. Determination of Minimum Inhibitory Concentration (MIC)

The multi-resistant strains of *Salmonella* Heidelberg, *Salmonella* Typhimuirium, *Salmonella* Typhimuirium var. monophasic 5,4,12:i:-, Avian Pathogenic *E. coli* (APEC) and *Pseudomonas aeruginosa* were inoculated on agar plates and incubated at 37 °C for 24 h [2]. The strain of *Campylobacter jejuni* was inoculated in CCDA-Preston agar (Oxoid) and incubated in microaerobic conditions for 48 h [21]. Isolated colonies were collected and re-suspended in 1 mL of sterile saline solution (0.9%) until the final concentration of 1.5 × 10^8^ CFU mL^−1^. The bacterial suspension was then diluted in 96-well plate wells to a final concentration of 1 × 10^5^ CFU·mL^−1^ per well (0.5 McFarland Standard).

Then, different concentrations of PCT-LIP/NOR and PCT@LIP/NOR were added in 96-well plates to a final volume of 0.1 mL. The positive control (NOR) was prepared for a final volume of 0.1 mL 1 × 10^5^ CFU·mL^−1^ of bacteria. The negative control was prepared with Muller Hinton Broth without bacteria (Oxoid^®^ Altrincham, Cheshire, UK), except for *Campylobacter jejuni*, in which the medium was supplemented with cation-adjusted (25 mg/L Ca^2+^, 12.5 mg/L Mg^2+^) solution and 5% lysed sheep blood (Laborclin^®^, Pinhais, Brazil), agreeing with ISO 20776-1 [22]. The 96-well plate of each bacterial strain was incubated at 37 °C for 24–48 h. The minimum inhibitory concentration (MIC) was determined for each sample. This experiment was performed in triplicate. ANOVA/Tukey were the statistical methods employed (*p* < 0.05).

### 2.8. In Vivo Toxicity Assays in Chicken Embryo Model

The in vivo toxicity of free NOR, PCT-LIP/NOR and PCT@LIP/NOR formulations was evaluated in chicken embryos through different parameters: changes in embryo weight/annexes and biochemical markers in the allantoic fluid: aspartate aminotransferase (AST), alanine aminotransferase (ALT), total antioxidant activity, reactive oxygen species (ROS), thiol group, superoxide dismutase (SOD) and catalase (CAT).

#### 2.8.1. Preparation of Eggs

The eggs of laying hens (*Gallus gallus*), lineage Hy-Line W-36 were donated by Hy-Line do Brazil (Uberlândia, Brazil). Pilot tests (see supplementary material) determined the suitable age of the embryos (Table S2) and NOR concentration (Table S3) to be employed in toxicity tests. Before analyses, the eggs are submitted to a light ovoscopy to ensure the quality and embryonic development. In a laminar flow, the eggs were disinfected and small holes were made in the center of the eggshell, enabling bacterial inoculation in the chorioallantoic membrane (CAM). Then, the eggs were incubated at 37.5 °C under 58% relative humidity (RH). Subsequently, 40 eggs with 10 embryos days old (ED) were identified, separately weighed and divided into 4 batches (*n* = 10) as follows: NC—negative control, eggs treated with deionized water; NOR—20 μg/mL norfloxacin; PCT-LIP- conventional liposomes blended with 0.5% pectin (*w*/*v*) and 20 μg/mL NOR; PCT@LIP- hybrid liposomes with 20 μg/mL NOR. Such doses were administered on the eggs CAM and the eggs were incubated at 37.5 °C under 58% RH. After 24, 48, 72, 96 and 120 h, the respective doses were re-administrated.

#### 2.8.2. Loss of Moisture and Weight of Embryos and Yolk Sac

After a week (ED 17 eggs), the eggs were weighed, and the eggshell was broken for the embryo mortality verification. The embryos were weighed, their annexes were removed, and the yolk sac (YS) was also weighed. The moisture loss was calculated by the difference between egg weight before (ED 10) and after (ED 17) treatments. The weight of embryos and YS were adjusted considering the initial weight (ED 10), using Equation (4):

(4) $$aW=\frac{\left(ce.ysW.50\right)}{ieW}$$

where *aW* is the weight adjusted to 50 g; *ce.ysW* is the chicken embryo or YS weight on ED 17, and *ieW* the initial egg weight on ED 10.

#### 2.8.3. Biochemical Analysis of Allantoic Fluid

The allantoic fluid (AF) of the embryos was collected and centrifuged at 1000× *g* 10 min. The supernatant was used to quantify the activity of alanine aminotransferase (ALT) and aspartate aminotransferase (AST) by the kinetic UV-IFCC (International Federation of Clinical Chemistry and Laboratory Medicine) method [23,24]. The biochemical analyses were processed in an automatic Labmax Plenno^®^ analyzer, using a Labtest Diagnóstica^®^ (Lagoa Santa, MG, Brazil) kit. The equipment was previously calibrated with Calibra 1 and standardized with Qualitrol 1 H universal control serum, both produced by Labtest Diagnóstica^®^.

#### 2.8.4. Biomarkers of Oxidative Stress

For the preparation of liver tissue, the chicken embryos were euthanized, and their liver were removed and immediately stored at −80 °C. The samples were homogenized with 10 mM sodium phosphate buffer (pH 7.4) and centrifuged at 800× *g* 4 °C for 10 min. The supernatant was used to quantify the biomarkers of oxidative stress.

#### 2.8.5. Catalase (CAT)

CAT activity determination was based on hydrogen peroxide decomposition by the enzyme present in the liver samples. The liver homogenates were incubated with 10% Triton X-100 and mixed with 10 mM potassium phosphate buffer (pH 7.0) containing 0.2% hydrogen peroxide. The hydrogen peroxide decomposition was monitored at 240 nm, during 10 min [25].

#### 2.8.6. Superoxide Dismutase (SOD)

SOD activity was assessed by the inhibition of auto-oxidation of pyrogallol by the enzyme present in the liver samples. The liver homogenates were mixed with 50 mM Tris-HCl buffer (pH 8.2) containing 1 mM EDTA, 80 μM catalase and 24 mM pyrogallol. The kinetics was monitored during 10 min, at 420 nm [26].

#### 2.8.7. Total Antioxidant Capacity

The liver homogenates were incubated with 300 mM sodium acetate buffer (pH 3.6), 10 mM 2,4,6-tri(2pyridyl)-striazine (TPTZ) and 20 mM ferric chloride at 37 °C for 6 min, at 593 nm. The antioxidant capacity was determined by a trolox analytical curve and sodium acetate buffer was used as blank [27].

#### 2.8.8. Reactive Oxygen Species (ROS)

The samples were incubated with dichloro-dihydro-fluorescein diacetate (10 µM) and 5 mM Tris-HCl buffer (pH 7.4) for 3 min. After, the fluorescence was measured at 530 nm (excitation in 474 nm).

#### 2.8.9. Lipid Peroxidation

The liver homogenates were incubated with 0.67% thiobarbituric acid (0.67% TBA) and 10% trichloroacetic acid (TCA), for 120 min. Then, *n*-butanol was added to the samples to remove the organic-phase and the fluorescence was measured at 553 nm, after excitation in 515 nm. The lipid peroxidation was determined using malondialdehyde (MDA) analytical curve [26].

#### 2.8.10. Thiol Group

Thiol group was detected using ditionitrobenzoic acid (DTNB) diluted in 0.2 mM potassium phosphate buffer (pH 8.0). The liver homogenates were incubated for 30 min with 1 mM phosphate buffer (pH 7.4) and 10 mM DTNB solution. The presence of sulfhydryl groups was spectrophotometrically detected at 412 nm [28].

## 3. Results

In this work we have prepared conventional (LIP/NOR) and hybrid (PCT-LIP/NOR and PCT@LIP/NOR) liposomes for the encapsulation of NOR, as well as their respective controls (without drug). The two hybrid systems differed regarding PCT distribution inside and outside the liposomes: in PCT-LIP, PCT is only found in the aqueous phase that surrounds the liposomes, while in PCT@LIP the biopolymer was distributed in the water phase inside and outside the vesicles. The antibiotic NOR was found in the exterior and interior of the vesicles in both systems (PCT-LIP/NOR and PCT@LIP/NOR). Moreover, since no buffer was used in the preparation of PCT@LIP/NOR (final pH = 4.8), the dissolution of NOR in the water phase was favored, as well as its electrostatic interaction with the anionic PCT biopolymer.

### 3.1. Physicochemical Stability of the Liposomes

**Figure 1:**
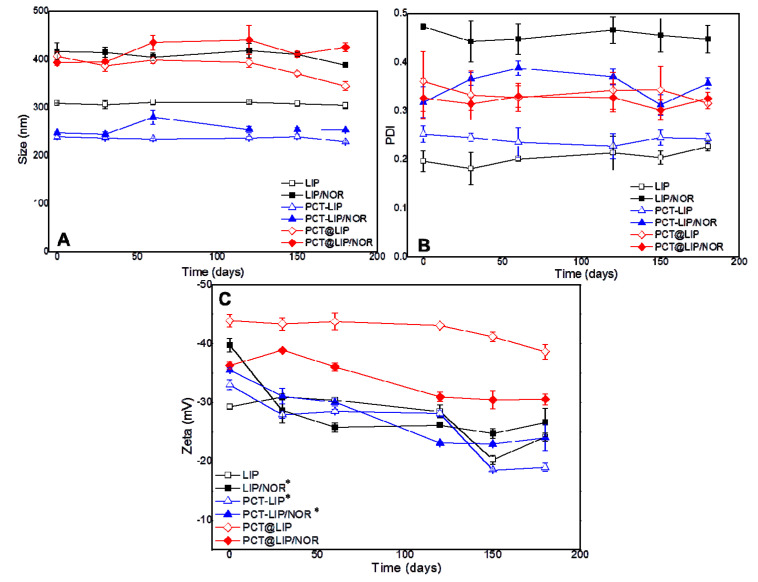
Stability study of the formulations with and without 0.2% NOR (*w*/*v*) in terms of size (**A**), PDI (**B**), and zeta potential (**C**) during 6 months of storage at 4 °C, *n* = 3. Statistical methods ANOVA/Tukey were used; * = *p* < 0.05.

The physicochemical stability of the vesicles was monitored in terms of size (nm), PDI and zeta potential (mV) assessed by DLS, for 180 days (Figure 1). Size (Figure 1A) ranged from 228–344 nm in all control liposomes, and from 253–425 nm in the liposomes containing 0.2% NOR (*w*/*v*). Regarding PDI (Figure 1B), all the samples exhibited values between 0.2 and 0.4 during the 6 months period. No statistically significant differences were observed neither in size (*p* > 0.05) nor in PDI (*p* > 0.05) over time. On the other hand, zeta potential values (Figure 1C) exhibited statistically significant differences (*p* < 0.05) after 180 days for LIP/NOR, PCT-LIP and PCT-LIP/NOR formulations, with relevant decrease regarding the initial values. PCT@LIP and PCT@LIP/NOR showed zeta values around −39 and −31 mV, respectively, at the end of the test, with no significant changes over time.

### 3.2. Morphology of Hybrid Liposomes

**Figure 2:**
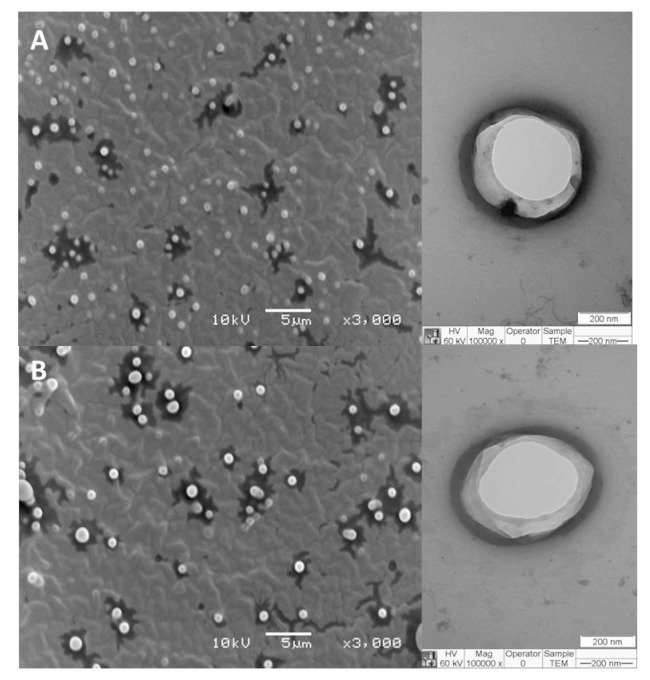
FE-SEM (left) and TEM (right) images of PCT@LIP (**A**) as control and PCT@LIP/NOR (**B**) containing 0.2% NOR (*w*/*v*) formulations. The used magnifications are displayed in the images.

The morphology of PCT@LIP and PCT@LIP/NOR formulations was assessed through TEM and MEV analyses (Figure 2). The hybrid formulations showed spherical shape with visible contours (Figure 2B) and narrow distribution (Figure 2A). The existence of both NOR and PCT in these systems did not disrupt the nanoparticles neither influence their sizes, since the vesicles size estimated by ImageJ software was around 380 and 425 nm for PCT@LIP and PCT@LIP/NOR, respectively, in agreement with the sizes measured by DLS (Figure 1).

### 3.3. Encapsulation Efficiency (%EE)

The encapsulation efficiency of 0.2% NOR (*w*/*v*) by the liposomal formulations was measured through the ultrafiltration/centrifugation method, in triplicate. The profile of the determined %EE was: PCT@LIP/NOR (83.7 ± 0.4) > PCT-LIP/NOR (63.1 ± 0.8) > LIP/NOR (61.3 ± 0.3).

### 3.4. In Vitro Release Test

**Figure 3:**
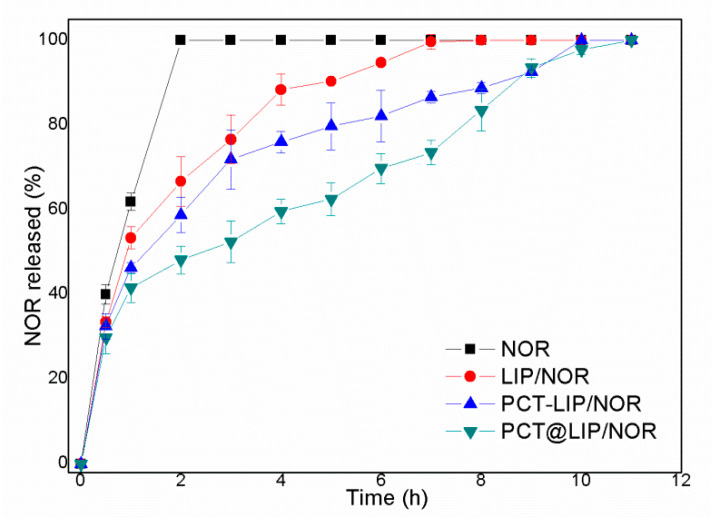
In vitro release profile of norfloxacin (0.2%), free or encapsulated in LIP/NOR, PCT-LIP/NOR, and PCT@LIP/NOR samples, at 37 °C, *n* = 5.

Figure 3 shows the release profile of commercial 0.2% NOR and liposomal formulations containing equivalent NOR concentration. After 2 h of experiment, the release from NOR, LIP/NOR, PCT-LIP/NOR and PCT@LIP/NOR reached 100, 67, 56 and 48%, respectively. Total NOR release (100%) was reached after 8, 10 and 11 h of experiment, for conventional liposomes (LIP/NOR), PCT-LIP/NOR and PCT@LIP/NOR hybrid systems, respectively.

Mathematical modeling (Table S1) was employed to fit the samples kinetics through different models (zero order, first order, Korsmeyer-Peppas, Higuchi and Weibull). Considering the highest coefficient of determination (R^2^) values, LIP/NOR and PCT-LIP/NOR were best fitted by Korsmeyer-Peppas suggesting an anomalous transport [29], while PCT@LIP/NOR was better described by the first-order model compatible with a biphasic release profile [20].

### 3.5. Determination of Minimum Inhibitory Concentration (MIC)

PCT-LIP/NOR and PCT@LIP/NOR formulations were selected for the antimicrobial and in vivo toxicity assays, based on their stability, %EE, and sustained release profile results. Table 1 displayed MIC of free and encapsulated 0.2% NOR (*w*/*v*), against several bacterial strains.

It was observed that both PCT-LIP/NOR and PCT@LIP/NOR showed higher bactericidal capacity (*p* < 0.05) (given by lower MIC) than commercial NOR, against all multi-resistant strains tested. For instance, MIC against *Pseudomonas aeruginosa* isolated from humans was >30 μg/mL for NOR and 3.2 μg/mL for PCT-LIP/NOR and PCT@LIP/NOR. In general, MIC ranged from 2 to 208 μg/mL for NOR, while in the hybrid formulations it varied from 0.2 to 80 μg/mL, for all bacterial strains tested.

### 3.6. In Vivo Nanotoxicity Test through Embryo Chicken Model

#### 3.6.1. Chicken Embryos Mortality, Weight of Embryos and YS

**Figure 4:**
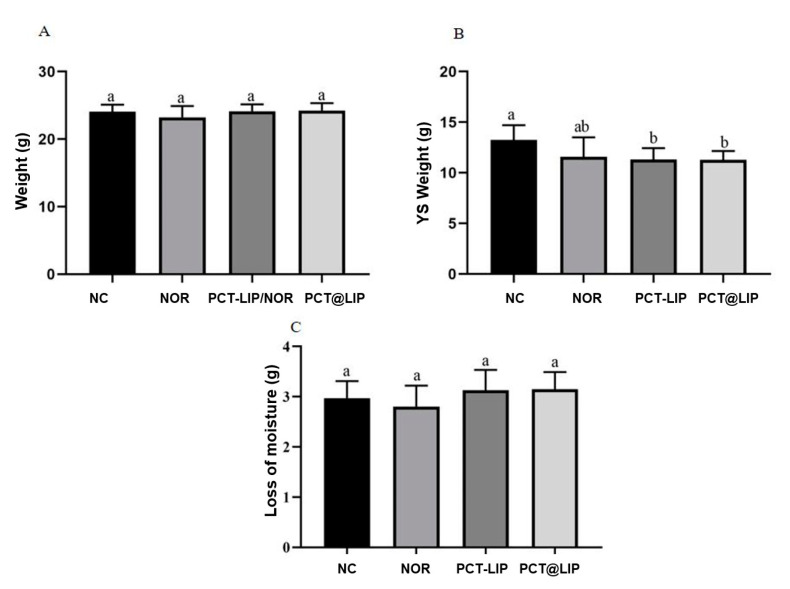
In vivo toxicity study in terms of chicken embryo weight (**A**), YS weight (**B**) and loss of moisture (**C**), in chicken embryos after treatment with norfloxacin (20 μg/mL) or formulations containing 20 μg/mL NOR. NC = negative control, NOR = 20 μg/mL norfloxacin solution, PCT-LIP = liposome encapsulating NOR (20 μg/mL) blended with pectin solution and PCT@LIP = pectin-liposome formulation containing NOR (20 μg/mL), *n* = 10. Different letters indicate significant difference (*p* < 0.05) and values are expressed as mean ± SEM (One-way ANOVA followed by Tukey test).

In all analyzed samples chicken embryos were alive on ED 17. There were no differences in the weight of the chicken embryos (Figure 4A) or in the loss of moisture between ED 17 and ED 10 (Figure 4C). However, the weight of the YS was less than the negative control for PCT-LIP/NOR and PCT@LIP/NOR (Figure 4B), with no statistically significant differences in comparison with free NOR.

#### 3.6.2. Levels of ALT and AST

**Figure 5:**
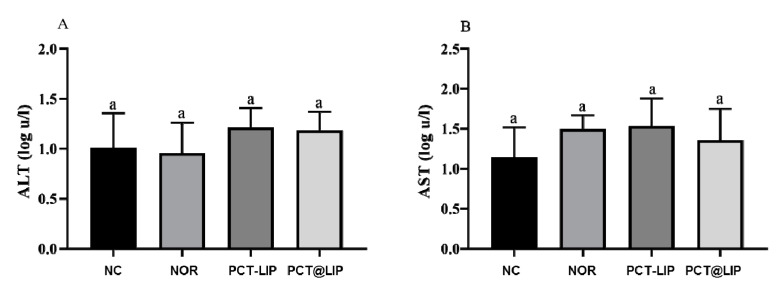
ALT (**A**) and AST (**B**) levels in AF of chicken embryos after treatment with free norfloxacin (NOR) or encapsulated in the formulations: PCT-LIP = conventional liposomes plus NOR at 20 μg/mL blended with pectin solution, PCT@LIP = pectin-liposomal formulation containing NOR at 20 μg/mL. NC = negative control, n = 10. Data expressed as mean ± SEM. Statistical test: One-way ANOVA/Tukey (*p* < 0.05).

Figure 5 shows that no statistically significant difference for ALT and AST activity was detected in the embryo’s allantoid fluid for any tested sample, in comparison to the negative control.

#### 3.6.3. Biomarkers of Oxidative Stress

**Figure 6:**
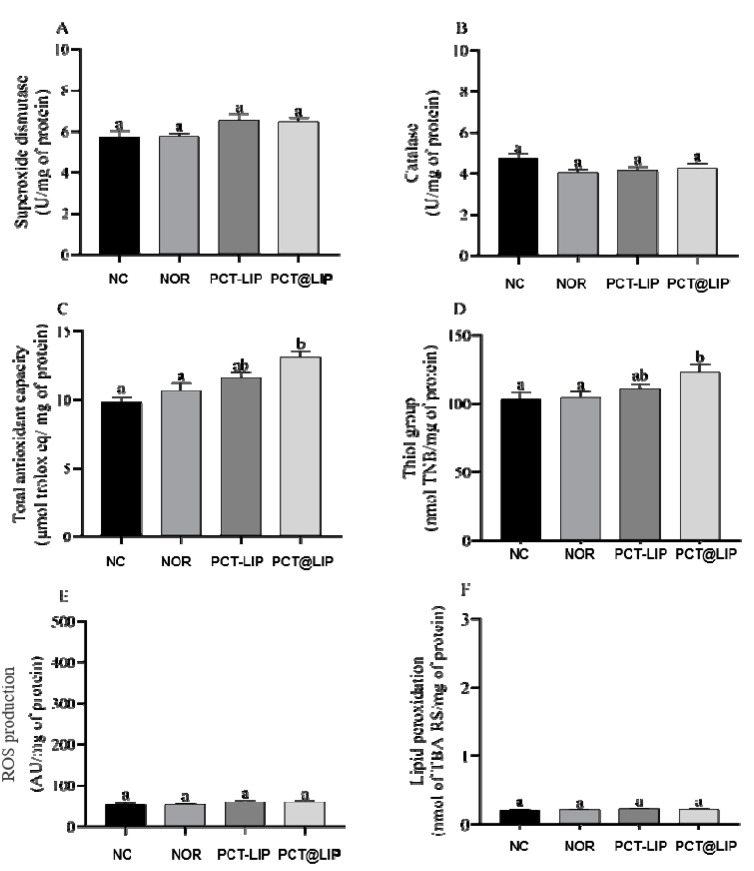
Levels of oxidative stress biomarkers in terms of superoxide dismutase (**A**), catalase (**B**), total antioxidant capacity (**C**), thiol groups (**D**), ROS production (**E**) and lipid peroxidation (**F**), in embryos liver chicken after treatment with norfloxacin (NOR, 20 μg/mL) or formulations containing 20 μg/mL NOR: PCT-LIP = liposomes encapsulating NOR and blended with pectin solution and PCT@LIP = hybrid liposomal-pectin formulation containing NOR. NC = negative control; n = 10. Data expressed as mean ± SEM. Statistical tests: One-way ANOVA/Tukey (*p* < 0.05).

Figure 6 shows the levels of SOD, CAT, ROS or lipid peroxidation in the AF of chicken embryos treated with NOR, PCT-LIP/NOR and PCT@LIP/NOR. In comparison to the controls, none of the treatments had significantly changed those parameters. It was also demonstrated that with exception of NOR, PCT-LIP/NOR and PCT@LIP/NOR formulations increased the levels of thiol groups (*p* < 0.05) and the total antioxidant capacity (*p* < 0.05) of the embryos, in relation to the negative control.

## 4. Discussion

Liposomes have been successfully described for the encapsulation of several antibiotics aiming different applications. These vesicles are composed of phospholipids that can be internalized by bacterial membranes [30], favoring the delivery of antimicrobial agents in situ. However, the huge increase of multi-resistant strains have encouraged the development of modified liposomes in order to increase the bactericidal effect of antimicrobial drugs [5], so that fusogenic and cationic liposomes containing vancomycin have been used against nosocomial and community-acquired infections [31].

Previous studies have shown evidences of NOR interaction with the lipid phase of model membranes, such as protection of NOR photodegradation when encapsulated in liposomes [8] or the increase in membrane fluidity in the presence of NOR [32]. However, the partition coefficient of NOR is low (0.46) [33], probably given by its amphoteric behavior at physiologic pH. Accordingly, when uploaded in conventional liposomes, NOR is expected to be found not only partitioned in the bilayer, but also in the aqueous phase of the vesicles [9,34]. In order to increase NOR upload from liposomes, it was hypothesized that if a nanocarrier was able to improve drug loading, this could have its therapeutic efficacy improved. Therefore, PCT@LIP/NOR hybrid formulation was prepared using two organic carriers, liposomes and pectin, that contributed with different structural organization and biological properties to the systems. NOR is an amphoteric molecule that at acidic pH interacts with PCT, so that the pH of the PCT@LIP/NOR formulation (4.8) favored drug upload, in comparison to PCT-LIP and conventional (LIP) formulations, prepared at pH 7.0 and pH 7.4, respectively.

All formulations were successfully prepared, showed a translucent appearance and spherical morphology (Figure 2), as previously observed with conventional liposomes of the same composition [35,36]. Encapsulation of NOR increased the size and PDI of conventional liposomes, which was not evident in PCT-containing formulations. The vesicles were physically stable for 6 months, and PCT@LIP/NOR was the formulation with highest zeta values in modulus (from the contribution of anionic superficial charges of pectin).

The higher NOR encapsulation efficiency in PCT@LIP/NOR than PCT-LIP/NOR resulted from the low pH of pectin (~4.5) solution [37] used to form the PCT@LIP vesicles. Since NOR is an amphoteric molecule, with ionizable quinolone carboxyl (pKa_1_ = 6.87) and piperazine nitrogen (pKa_2_ = 8.75) groups, the protonation of the carboxylic groups of NOR under acidic pH eliminates a negative charge, and favors the interaction between its protonated amine groups with the carboxylic groups of PCT in solution. Indeed, the highest %EE also explains the longest sustained release profile of NOR and the most pronounced in vitro antimicrobial activity of PCT@LIP/NOR, among all formulations tested.

The sustained release of NOR was prolonged for 11 h for PCT@LIP/NOR. In agreement to this, Giovanni and coworkers prepared liposomes composed of egg phosphatidylcholine/cholesterol/α-tocopherol loading fluroquinolone antibiotics at pH 7.4. In the in vitro release test, ofloxacin-loaded liposomes released around 90% after 10 h of experiment [9]. In another report, specialized (fusiogenic and cationic) liposomes composed of phosphatidylcholine/cholesterol/α-tocopherol released around 70% vancomycin after 10 h [31]. In addition, the acidic liposomes containing PCT in their inner aqueous phase (PCT@LIP/NOR) have driven a different release kinetic profile (first order) of NOR in comparison to LIP/NOR and PCT-LIP/NOR (Korsmeyer-Peppas), corroborating its more complex supramolecular structure. Considering that NOR release profile from PCT@LIP/NOR was best described by the first-order model, it means that the kinetics was governed by two phases, an initial given by the erosion of biopolymer matrix containing NOR (outside the vesicles), followed by the sustained drug diffusion from the liposomes, where the drug had to overcome two physical barriers to be released: the lipid bilayer and polysaccharide network, as also described for carboxymethyl-chitosan coated liposomes, xanthan-lipid nanoparticles hydrogels and pectin-lipid nanofilms [20,38,39].

In the antimicrobial tests, it is worth mentioning that the strains tested here are difficult to handle, including multi-resistant *Pseudomonas aeruginosa* isolated from dead humans and APEC (avian pathogenic *E. coli*) isolated from dead chickens. The abovementioned results suggest that both hybrid pectin-liposomal formulations were efficient to treat multi-resistant bacterial infections, possibly in lower doses than commercial NOR. Moreover, the nanostructured vesicles provided a huge external surface area for the specific interaction with the bacterial cell wall [5], enhancing the release and antimicrobial activity of NOR. In addition, PCT is widely applied as a gastroprotective matrix, due to its resistance to acidic pH [15] and as a mucoadhesive excipient, for the ability to interact with the carboxylic acid of mucin [40]. PCT@LIP/NOR combined quite interesting properties of both liposomes and the biopolymer carriers. This system can also be processed as different pharmaceutical forms for several routes of administration, minimizing the side effects of traditional antibiotic therapy and improving the patient compliance to the treatment.

Another essential parameter to ensure that a nanostructured formulation can reach the market is its safety [10]. There are several in vitro and in vivo models to evaluate the biocompatibility of pharmaceutical forms [41]. Alternative in vivo toxicity models are currently employed to verify the toxicity of formulations, drugs and natural compounds, such as the zebrafish [42,43] and chicken embryo [44] assays.

The safety of PCT-LIP/NOR and PCT@LIP/NOR was carried out using in vivo chicken embryo [44] model, analyzing several parameters and biochemical markers. Although fluoroquinolones can induce adverse effects on avian embryonic development, reducing the performance of incubated eggs and hatching chicks [45], PCT-LIP/NOR and PCT@LIP/NOR did not cause a decrease in the weight of the chicken embryos, increase in moisture loss of eggs or embryo mortality. In fact, neither of the commercial NOR induced any such chicken embryo damaged alterations. Once the liposomal formulations optimized NOR antimicrobial activity, its biocompatibility was not expected to be affected, as observed here. On the other hand, free NOR, PCT-LIP/NOR and PCT@LIP/NOR caused a significant decrease of YS weight in comparison to the negative control, with no significant statistic differences between any liposomal formulation and free NOR. Such results were expected, once fluoroquinolones administration can result in inappropriate yolk formation in chicken embryos [46]. It is worth mentioning that the amount of drug used in the antimicrobial tests (20 μg/embryo) was not able to evoke any in vitro antimicrobial activity for commercial NOR, while MIC_50_ for PCT@LIP/NOR ranged from 0.5 to 13 μg/mL, against all the bacterial strains tested, enabling further safety treatments with lower doses when compared with commercial drug.

Moreover, the quantification of ALT and AST activities, total antioxidant activity, ROS, thiol groups, SOD and CAT were also performed in the liver tissue.

The alteration of physiological enzymes activity can show if a compound is toxic or not to biological tissues, increases in the activity of ALT and AST being specifically related to liver injury [44]. None of the formulations increased the levels of ALT and AST in relation to controls, suggesting that there was no liver damage in the embryos, after treatment.

The generation of ROS in cells is also important, once they act as mediators for the transfer of electrons in the biochemical reactions in the body. However, excessive ROS production in cells can lead to oxidative damage, affecting organs functions in different ways [47]. Consequently, cells use antioxidant mechanisms in order to prevent cell damage [48], and the quantification of oxidative stress biomarkers can assess liver side effects [49]. Here, the embryo treatment with the formulations did not induce increase of the production of ROS, CAT and SOD activity or lipid peroxidation in comparison to controls, as required for biocompatible systems.

Interestingly, there was a statistically significant increase (*p* < 0.05) in the total antioxidant capacity and thiol groups levels in the embryos treated with liposomal formulations in comparison to free NOR and the negative control. This evidenced an additional advantage of these hybrid systems in relation to the commercial drug. Such antioxidant effect can be explained by the presence of α-tocopherol in the composition of liposomes, since this vitamin prevents the peroxidation of EPC, the major component of the liposomes that is polyunsaturated [50]. α-tocopherol is a highly lipophilic antioxidant molecule against oxidative stress and also can act as a protector of thiols groups present in many proteins and in reduced glutathione [51,52,53]. According to the abovementioned results, PCT@LIP/NOR is ready to be further tested in efficacy assays against multidrug resistant bacteria and clinical trials.

## 5. Conclusions

The hybrid pectin-liposomal formulation for NOR delivery, composed of PCT@LIP/NOR, showed physicochemical stability for 180 days of storage at 4 °C, desirable structural properties, prolonged the release profile of NOR for 11 h (9 h more than commercial NOR) and remarkable antimicrobial activity against different multi-resistant bacterial strains. The safety of hybrid formulations was ensured, based on different parameters and the quantification of biochemical markers, through an in vivo embryo chicken model. PCT@LIP/NOR will be promising for the treatment of multidrug-resistant bacterial infections of interest in public health and veterinary clinic, allowing higher bioavailability and lower side effects than conventional antibiotic therapy, through the administration of lower doses and improving the patient compliance to the treatment.

## Figures and Tables

**Table 1:** Determination of the minimum inhibitory concentration (MIC, μg/mL) for NOR (in solution), PCT-LIP/NOR and PCT@LIP/NOR formulations against different bacterial strains; *n* = 4.

Samples	SH	ST var	ST	CJ	PA	*E. coli*
NOR	208.0 ± 72.0	125.0 ± 0.0	208.0 ± 72.0	2.0 ± 0.0	>30	30.0 ± 0.0
PCT-LIP/NOR	84.0 ± 3.0 *	32.0 ± 27.0 *	31.0 ± 0.0 *	0.2 ± 0.0 *	3.2 ± 0.0 *	3.2 ± 0.0 *
PCT@LIP/NOR	5.0 ± 3.0 *	4.0 ± 3.0 *	13.0 ± 5.0 *	0.5 ± 0.0 *	3.2 ± 0.0 *	6.1 ± 2.4 *

Note: SH = *Salmonella* Heildelberg, ST var = *Salmonella* Typhimuirium var. monophasic 5,4,12:i:-, ST = *Salmonella* Typhimuirium, CJ = *Campylobacter jejuni*, PA = *Pseudomonas aeruginosa*, *E. coli* = *Escherichia coli* isolated from humans. * Indicates statistically significant difference between PCT-LIP/NOR or PCT@LIP/NOR and free NOR against each strain; *p* < 0.05 (unpaired *t*-test).

## Supplementary Materials

The following are available online at https://www.mdpi.com/1999-4923/12/8/769/s1. Figure S1: Chemical structure of norfloxacin, C16H18FN3O3, Molecular Weight 319.33 g/mol, Figure S2: Chemical structure of pectin monomer, C6H10O7, Molecular Weight 194.14 g/mol. Table S1. Mathematical modeling of Kinetic curves from the samples according to the determination coefficient (R2) values. Table S2. Number of surviving embryos (after 24 and 48 h) infected (via CAM) with different concentrations of Salmonella Heidelberg, on ED 8, 10 and 14. Table S3. Number of surviving embryos (after 48 h) infected (via CAM) with different concentrations of Salmonella Heidelberg, on ED 8 and10, treated or not with NOR (2, 20 and 50 μg/embryos).
