# Supplementary material for: Hybrid Pectin-Liposome Formulation against Multi-Resistant Bacterial Strains
Source: Pharmaceutics. 2020 Aug 14;12(8):769. doi: 10.3390/pharmaceutics12080769 (PMC7465986; doi:10.3390/pharmaceutics12080769)
Supplement: Supplementary file 1 [file pharmaceutics-12-00769-s001.pdf]

pharmaceutics

MDPI

Supplementary Materials: Hybrid Pectin-Liposome Formulation against Multi-Resistant Bacterial Strains

Lígia Nunes de Morais Ribeiro *, Eneida de Paula, Daise Aparecida Rossi, Guilherme Paz Monteiro, Edson Campos Valadares Júnior, Rogério Reis Silva, Rodrigo Franco, Foued Salmen Espíndola, Luiz Ricardo Goulart and Belchiolina Beatriz Fonseca

Introduction

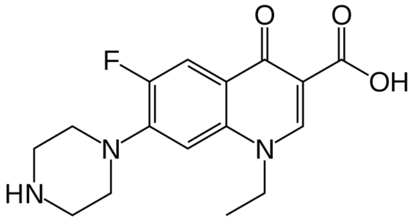

Figure S1. Chemical structure of norfloxacin, $C_{16}H_{18}FN_3O_3$, Molecular Weight 319.33 g/mol.

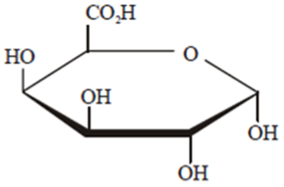

Figure S2. Chemical structure of pectin monomer, $C_6H_{10}O_7$, Molecular Weight 194.14 g/mol.

Methods

In vivo Toxicity Assays in Chicken Embryo Model

Pilot Test

A total of 75 eggs were used to inoculate different doses of *Salmonella* Heidelberg (1, 2, 3 and 5_{log}CFU/embryo) on CAM (*n* = 5), to verify the minimum bacterial concentration that kill the embryos with different ED (8, 10 and 14) (table S1). The embryo mortality was evaluated after 24 and 48 h by ovoscopy. In this test, the minimum amount of Salmonella that kill the chicken embryo was 2_{log}UFC/embryo. Therefore, 2_{log}UFC/embryo of ED 8 and 10 were employed in the subsequent analyses.

Then, it was determined the lowest dose of NOR (2, 20 or 50 µg/embryo) capable of saving the embryo after 1 h of Salmonella infection (table S2). ED10 embryos whit 20 µg/embryo NOR were selected to carried out the toxicity test.

Table S1. Mathematical modeling of Kinetic curves from the samples according to the determination coefficient (R^2) values.

Formulations	0 Order	First Order	Higuchi	Korsmeyer-Peppas	Weibull
LIP/NOR	0.76	0.66	0.49	0.94	0.79
PCT-LIP/NOR	0.88	0.77	0.69	0.98	0.60
PCT@LIP/NOR	0.63	0.93	0.81	0.86	0.46

Note: LIP/NOR = liposome encapsulating (0.2%) NOR, PCT-LIP= liposome encapsulating (0.2%) NOR blended with pectin solution and PCT@LIP= pectin-liposome formulation containing (0.2%) NOR, *n* = 5.

pharmaceutics

MDPI

Table S2. Number of surviving embryos (after 24 and 48 h) infected (via CAM) with different concentrations of *Salmonella* Heidelberg, on ED 8, 10 and 14.

ED	***Salmonella* Heidelberg ($_{log}$CFU/embryo)** NC		2		3		4		5	
	24 h	***48 h***	*24 h*	*48 h*	*24 h*	*48 h*	*24 h*	*48 h*	*24 h*	*48 h*
8	5	5	5	5	5	5	5	5	5	5
10	5	5	0	5	0	5	0	5	0	5
14	5	5	5	5	5	5	5	5	5	5

Note: NC – Negative control, eggs treated with deionized water; ED - embryos days old.

Table S3. Number of surviving embryos (after 48 h) infected (via CAM) with different concentrations of *Salmonella* Heidelberg, on ED 8 and10, treated or not with NOR (2, 20 and 50 μg/embryos).

ED	NC	Sal	NOR 2	NOR 20	NOR 50	Sal/NOR 2	Sal/NOR 20	Sal/NOR 50
**8**	5	0	0	0	0	0	0	0
**10**	5	0	5	5	5	0	5	5

Note: NC: negative control, eggs treated with water; Sal: positive control, inoculation with Salmonella; NOR 2: eggs treated with 2 μg/embryo norfloxacin; NOR 20: 20μg/embryo norfloxacin; NOR 50: 50μg/embryo norfloxacin; Sal/NOR 2: eggs inoculated with 2$_{log}$UFC Sal and treated with 2μg/embryo NOR after 1 h; Sal/NOR 20: eggs inoculated with 2$_{log}$UFC Sal and treated with 20μg/embryo after 1 h; Sal/NOR 50: eggs inoculated wit 2$_{log}$UFC Sal and treated with 50μg/embryo after 1h.
